# Low Molecular Weight (poly)Phenol Metabolites Across the Blood-Brain Barrier: The Underexplored Journey

**DOI:** 10.3233/BPL-200099

**Published:** 2021-02-09

**Authors:** Rafael Carecho, Diogo Carregosa, Cláudia Nunes dos Santos

**Affiliations:** a CEDOC, NOVA Medical School, Faculdade de Ciências Médicas, Universidade NOVA de Lisboa, Campo dos Mártires da Pátria, Lisboa, Portugal; b iBET, Instituto de Biologia Experimental e Tecnológica, Avenida da República, Apartado 12, Oeiras, Portugal; c Instituto de Tecnologia Química e Biológica António Xavier, Universidade NOVA de Lisboa, Avenida da República, Oeiras, Portugal

**Keywords:** Polyphenols, metabolites, BBB, microbiota, brain, permeability

## Abstract

The world of (poly)phenols arising from dietary sources has been significantly amplified with the discovery of low molecular weight (LMW) (poly)phenol metabolites resulting from phase I and phase II metabolism and microbiota transformations. These metabolites, which are known to reach human circulation have been studied to further explore their interesting properties, especially regarding neuroprotection. Nevertheless, once in circulation, their distribution to target tissues, such as the brain, relies on their ability to cross the blood-brain barrier (BBB), one of the most controlled barriers present in humans. This represents a key step of an underexplored journey towards the brain. Present review highlights the main findings related to the ability of LMW (poly)phenol metabolites to reach the brain, considering different studies: *in silico*, *in vitro,* and *in vivo.* The mechanisms associated with the transport of these LMW (poly)phenol metabolites across the BBB and possible transporters will be discussed. Overall, the transport of these LMW (poly)phenol metabolites is crucial to elucidate which compounds may exert direct neuroprotective effects, so it is imperative to continue dissecting their potential to cross the BBB and the mechanisms behind their permeation.

## (POLY)PHENOLS METABOLISM AND DISTRIBUTION

Phenolic compounds and polyphenols, commonly referred to as (poly)phenols, constitute a group of small molecules widely spread mostly in plants, being found in many fruits and vegetables. Their structures having at least one hydroxyl group and one aromatic ring, together with functional groups have been linked to a huge variety of beneficial properties to human health, some of them related with brain health [1–3].

Since they easily reach our organism through our daily diet, their impact on human’s health may be relevant. Therefore, to reveal their potential effects, several studies focused on understanding their routes after being ingested, the metabolism they are submitted, the distribution to reach the target tissues, and ultimately, the mechanisms behind their action. It has been established that the (poly)phenols influence the brain by modulating receptors function, interacting with neuronal signaling pathways, and by promoting the expression of proteins mainly involved in synaptic plasticity and neuronal repair [1–3]. The outcomes emerged from this research allow the scientific community to state the bioavailability and bioactivity of many dietary phenolic, especially flavonoids, and explore their role in several chronic diseases [1–3].

Importantly, dietary (poly)phenols are subjected to several biotransformations after oral intake. Phase I and phase II enzymes are responsible for intensive catabolism over the parent compounds both in the intestine and in the liver, leading to a broad range of (poly)phenol metabolites [1, 3, 4]. Moreover, they may undergo further metabolism in the colon by the gut microbiota into simpler compounds to be absorbed [5, 6]. All (poly)phenols, from simple phenolics to more complex ones, are submitted to this biotransformation. As result, the molecules that reach circulation and are able to influence the tissues are not anymore the same complex molecules present in the food consumed, but novel metabolites that have arisen from the gastrointestinal tract metabolism [1, 3, 7–9]. Along time, many mechanistic studies have been reporting the molecular effects on cellular models of isolated parent compounds without considering the processes of absorption and the metabolic reactions that (poly)phenols undergo within the human body. From those studies, some conclusions have been drawn that may not fit with results *in vivo*, where the influence of the described metabolic events over the compounds occurs.

Overall, colonic microbiota and liver metabolism originates a huge variety of metabolites. Some of them are unique metabolites derived from a specific parent compound (e.g. urolithins from ellagitannins, S-equol from isoflavones) [9, 10] but the majority of them, derived possibly from several parent compounds, are simpler metabolites named low-molecular-weight (LMW) (poly)phenol metabolites (Table 1). These LMW (poly)phenol metabolites are considered to reach circulation at considerably higher concentrations (10–30*μ*M) than their parent compounds [1, 9].

**Table 1:** LMW (poly)phenol metabolites disposition within human organism and pathways that are affect by those compounds. LMW (poly)phenol metabolites considered were detected in humans circulating in plasma or excreted in urine after dietary flavonoids ingestion^1^

Compound class	Common name	Organs detected (HMD)^2^	Pathways that could be involved (KEGG)^3^
**Benzene diols and triols**			
1,2-dihydroxybenzene derivatives	Catechol	Intestine/Bone marrow/Brain/Liver/Prostate	Predicted to be involved in COMT activity in the metabolism of catecholamine neurotransmitters and catechol hormones
	catechol-*O*-sulfate	–	–
	4-methylcatechol	–	–
	4-methylcatechol-*O*-sulfate	–	–
	Guaiacol	–	Predicted to be involved in COMT activity in the metabolism of catecholamine neurotransmitters and catechol hormones
	guaiacol-*O*-sulfate	–	–
1,3-dihydroxybenzene derivatives	Resorcinol	–	–
	resorcinol-*O*-sulfate	–	–
1,2,3-trihydroxybenzene derivatives	Pyrogallol	–	COMT inhibitor
	pyrogallol-*O*-sulfate	–	–
	pyrogallol-1-*O*-glucuronide	Liver/Kidney	–
	1-*O*-methylpyrogallol	–	–
	1-*O*-methylpyrogallol-*O*-sulfate	–	–
	2-*O*-methylpyrogallol	–	–
2-*O*-methylpyrogallol-1-*O*-sulfate	–	–
1,3,5-trihydroxybenzene derivatives	phloroglucinol-*O*-sulfate	–	Phloroglucinol is described as COMT inhibitor
	trimethoxyphloroglucinol	–	–
**Benzaldehydes**			
monohydroxybenzaldehydes	4-hydroxybenzaldehyde	–	–
dihydroxybenzaldehydes	Protocatechaldehyde	–	–
	Vanillin	–	–
trihydroxybenzaldehydes	Phloroglucinaldehyde	–	–
**Benzoic acids**			
Benzoic acid derivatives	benzoic acid	Testicle/Bladder/Liver/Skin/Kidney	–
	salicylic acid	Liver/Skin	–
	3-hydroxybenzoic acid	–	–
	4-hydroxybenzoic acid	Cerebrospinal Fluid	–
	benzoic acid-4-*O*-glucuronide	Kidney/Liver	–
dihydroxybenzoic acid derivatives	protocatechuic acid	Testicle
	protocatechuic acid-*O*-sulfate	–
	protocatechuic acid-*O*-glucuronide	–
	veratric acid	–
	isovanillic acid	–
	isovanilic acid-3-*O*-sulfate	–
	isovanilic acid-3-*O*-glucuronide	–
	vanillic acid	–	–
	vanillic acid-4-*O*-sulfate	–	–
	vanilic acid-4-*O*-glucuronide
Dihydroxybenzoic acids	2,4-dihydroxybenzoic acid	–	–
	gentisic acid	–	Product of tyrosine metabolism
	2,6-dihydroxybenzoic acid	–	–
	3,5-dihydroxybenzoic acid	–	–
3,4,5-trihydroxybenzoic acid derivatives	gallic acid	–	–
	3-*O*-methylgallic acid	–	–
	4-*O*-methylgallic acid	–	–
	4-*O*-methylgallic acid-3-*O*-sulfate	–	–
	syringic acid	–	–
	ethyl gallate	–	–
**Phenylacetic acids**			
Phenylacetic acid	phenylacetic acid	Cerebrospinal Fluid	Product of phenylalanine metabolism
Hydroxyphenylacetic acid	2-(2-Methoxyphenyl)acetic acid	–	–
	2-(3-hydroxyphenyl)acetic acid	–	Product of phenylalanine metabolism
	2-(4-hydroxyphenyl)acetic acid	Bladder/Epidermis/Kidney/Prostate	Product of tyrosine Metabolism
Dihydroxyphenylacetic acid	3,4-dihydroxyphenylacetic acid or homoprotocatechuic acid or DOPAC	Brain	Product of dopamine metabolism by monoamine oxidase (MAO) and further metabolized by COMT
	homovanillic acid	Brain/Central Nervous System/Kidney/Spinal Cord	Product of dopamine metabolism after DOPAC metabolism by COMT
**Mandelic acids**			
Mandelic acid	mandelic acid	–	–
Hydroxymandelic acid	4-hydroxy-mandelic acid	Retina/Vitreous humor	–
Dihydroxymandelic acid	Vanillylmandelic acid	Adrenal Gland/Epidermis/Thyroid Gland	Product of tyrosine metabolism
**Hippuric acids**			
Benzoylglycine	hippuric acid	Kidney/Liver/Prostate	Product of phenylalanine metabolism
Hydroxy Benzoylglycine derivatives	3-hydroxyhippuric acid	–	–
	4-hydroxyhippuric acid	–	–
**Cinnamic acids**			
	Cinnamic acid	Cinnamic acid	Liver
Hydroxycinnamic acids	*m*-coumaric acid	–	–
	*p*-coumaric acid	–	–
	*o*-coumaric	–	–
Dihydroxycinnamic acids	caffeic acid	Prostate	–
	isoferulic acid	–	–
	ferulic acid	–	–
	sinapic acid	–	–
**Phenylpropionic acids**			
Hydroxyphenylpropionic acid derivatives	3-hydroxyhydrocinnamic acid	–	–
	4-hydroxyhydrocinnamic acid (phloretic acid)	Kidney/Liver/Spleen	–
Dihydroxyphenylpropionic acid derivatives	dihydrocaffeic acid	–	–
	dihydroferulic acid	–	–
	dihydroisoferulic acid	–	–
**Phenylhydracrylic acid**			
3-(3-Hydroxyphenyl)-3-hydroxypropionic acid	3-(3’-Hydroxyphenyl)hydracrylic acid	–	–

^1^For more details concerning the presence in plasma or excreted in urine of the low molecular weight phenolic metabolites in humans after dietary flavonoids ingestion see review [9]. ^2^The Human Metabolome Database (HMDB) (http://www.hmdb.ca/) was used for searching available information concerning metabolites source (endogenous or by exposure) and the organs were have being described to be detected [13]. ^3^KEGG (Kyoto Encyclopedia of Genes and Genomes) database(https://www.kegg.jp/kegg/) was used to localize the metabolite in the KEGG pathways using the organism-specific pathway: Homo sapiens (human) [12]. The presence of the enzymes in humans was confirmed in BRENDA database (https://www.brenda-enzymes.org) [16].

Concerning the effect of (poly)phenol metabolites on brain function, it is still unclear whether their effect derives from direct action on brain cells or/and an indirect mechanism by affecting peripheral and cerebrovascular blood flow [2]. Considering the abundance of the LMW (poly)phenol metabolites and their relatively small structures, it is plausible to consider that these compounds could play the main roles in the direct effect on brain cells. Bearing this in mind, we previously reviewed the molecular mechanisms underlying LMW (poly)phenol metabolites effects, describing the scientific evidence of the bioactivity of these metabolites in the brain and their specific role on neuroinflammation [9]. The current review will focus on the data available for the brain bioavailability of these human LMW (poly)phenol metabolites, including evidence of crossing BBB *in silico*, *in vitro,* and *in vivo*.

Before emphasizing the data related to brain bioavailability of LMW (poly)phenol metabolites derived from the diet, it is important to state all possible origins of these compounds within the human body and the implication for brain function. Some of the gut microbes and host enzymes involved in the described biotransformations are responsible for the general metabolism of any chemical substance not naturally produced by or expected to be present within the human organism (xenobiotic metabolism). Therefore, it is not surprising that these LMW (poly)phenol metabolites could be obtained in humans not only through the regular diet, but also by metabolizing other ingested compounds, like residues of industrial chemicals and pharmaceuticals [11].

In fact, many of these compounds appear described as intermediates of xenobiotics biodegradation and metabolism pathways like the bacterial aminobenzoate degradation pathway [12]. On the other hand, it is also known that some of these compounds could appear in human circulation due to endogenous origin, due to the overlap with endogenous pathways such as the tyrosine or dopamine pathways [12].

Regardless of the mode of occurrence within the human body (by endogenous production or by dietary exposure to parent compounds) for some LMW (poly)phenol metabolites their presence in different human organs is already described in the human metabolome database (Table1) [13]. Moreover, since some of them are endogenously produced, we could expect a biological function in a specific pathway as indicated in Table 1. In fact, for some of these metabolites we highlighted the pathways where they could be involved, using the KEGG pathways database [12]. Additionally, since these metabolites share chemical similarities with some endogenous compounds it is also expected that some could interfere in the same pathways. Benzene diols metabolites due to their catechol moiety appear mentioned as involved in the metabolism of catecholamine neurotransmitters and catechol hormones, as substrate or products from catechol-O-methyltransferase (COMT) activity [12], while pyrogallol and phloroglucinol are inhibitors of the same enzyme [12, 14, 15]. Phenylacetic acids are mainly involved in aminoacid metabolism, phenylalanine, and tyrosine metabolism and as by-products of dopamine metabolism. Consequently, this is also the class of phenolic metabolites with more evidences in the human metabolome database of being detected in human brain [13]. Surprisingly, there is scarce data concerning their disposition within human organs and pathways that could be involved (Table 1).

Notably, the ability of metabolites in circulation to reach the target tissues is very important when considering their final effect. Their distribution relies on their capability to cross several barriers they may encounter during their journey. Regarding the nervous system, they will face one of the most regulated and controlled barriers in the human body, the blood-brain barrier (BBB), that must be crossed to directly influence the brain [17].

## BBB AND BBB DYNAMICS

BBB is a complex and highly selective semi permeable barrier found in most of the blood vessels that irrigate the brain, acting as a border between the circulating blood and the extracellular space of the central nervous system (CNS) [18]. It is formed by multiple cell types and proteins that ensure tight regulation of the movement of ions, molecules, and cells between the blood and the brain. This regulation allows controlling the accessibility of nutrients and molecules needed for brain homeostasis. It also prevents the entrance of toxins, pathogenic agents, and other harmful molecules that could impair the brain homeostasis. However, in an increased state of inflammation like in chronic diseases, BBB becomes more permeable, allowing the infiltration of viruses and bacteria. For the BBB to work properly, it requires the presence of tight junctions, which are central components of the barrier structure. Tight junctions maintain the endothelial cells attached to each other and restrict the passage by diffusion across the blood vessels [19]. Also, the astrocytes have an important role by projecting their end-feet to the walls of blood vessels, as well as the pericytes, which are involved in both endothelial cells support and paracrine signaling [19, 20].

Having this in mind, phenolic metabolites that reach the systemic circulation towards neuronal tissue are faced with this selective barrier that separates them from the brain. Meaning that, crossing the BBB represents a crucial step for metabolites in order to directly affect our brain.

In fact, this issue is not fully understood, and the mechanisms by which phenolic metabolites permeate the BBB are still unknown. The structure of metabolites should affect the effective uptake of these metabolites by the brain. Also, the routes used to access the brain might differ due to the structural differences of metabolites. Youdim et al showed that permeation across the BBB is associated with lipophilicity, meaning polar metabolites like sulfate and glucuronide conjugates will have reduced passage through the BBB by comparison with less polar methylated conjugated [21, 22]. Thus the degree of lipophilicity/polarity of each compound is a key factor that will determine transport by simple diffusion. Nevertheless, the primary route by which (poly)phenols cross the BBB: diffusion or specific carrier-mediated transport, is still not clear [23]. Moreover, the specific efflux transporters expressed in endothelial cells of BBB should be considered. These transporters may decrease the apparent permeability of the BBB to metabolites by transporting them back to the blood stream [24]. The question remains, however, whether the effects of phenolic metabolites on the modulation of brain functions are mediated directly in the brain or can be caused by peripheral cells as well since both scenarios are plausible. Additionally, recent studies have been reported the ability of endothelial cells to metabolize the phenolic metabolites into novel compounds before allowing their penetration into the brain [25]. Concerning this, more attention must be paid to the post-absorption events and intracellular metabolic reactions. This will allow us to distinguish what bioactive compounds are capable of affecting brain cells by modulating neuronal signaling pathways. As the evidence of anti-inflammatory properties attributed to (poly)phenols in the brain increases (reviewed in [9, 26, 27]), the focus turned to their capacity to cross the BBB, and different approaches from *in silico*, cell-based, and *in vivo* assays have been used to address that issue.

## TRANSPORT OF LMW (POLY)PHENOL METABOLITES THROUGH THE BBB

### In silico predictions of passive permeation

Although the presence of the LMW (poly)phenols metabolites has been confirmed in circulation as mentioned, the mechanisms by which these molecules cross the cell membranes into circulation and reach target organs are still quite underexplored, especially in the case of the brain. Several types of membrane transport have been considered for (poly)phenols. For flavonoids, several mechanistic studies on their transport have been proposed, due to their molecular weight and other chemical properties like polar surface and number and position of the oxygen atoms among others discussed below. The main idea of a transport, based on passive transport, has been substituted by the concept of flavonoids transport be mediated by ATP-binding cassette transporters (ABC) and organic anion transporters (OAT) [3] and also organic anion transporting polypeptides (OATP) [28, 29]. However, in the case of LMW (poly)phenol metabolites, the facts about their transport is still largely unknown.

Considering that the transport of (poly)phenol metabolites across cell membranes is known to be either through passive permeation, or active transport [23, 30, 31], this could potentially be extended to LMW (poly)phenol metabolites as well. Never the less, in the case of small molecules, defined as <500 g/mol, and their potential transport through BBB, one of the most studied possibilities is passive permeation [25, 32–34]. Passive permeation depends on the physical-chemical properties of a molecule and could be predicted by *in silico* methods. Yet, a limited number of studies have been conducted on LMW (poly)phenol metabolites molecules. These methods rely on physical-chemical parameters denominated by molecular descriptors, that are intrinsic to a molecule, for example molecular weight, number of hydrogen acceptor and donor bounds, volume, dipole moment, and many others [32]. All these parameters can then be conjugated into a single property designated commonly as log BB that represents the logarithmic ratio between the concentration of a compound in the brain and blood. This log BB property can then be used to compare between a library of known molecules that cross the BBB and the potential molecules to predict. Many of these models are present in the literature and are usually associated with drug discovery [35]. Due to the different molecular descriptors used for each model, a direct comparison between log BB cannot be made and are usually model- dependent. The ability of several LMW (poly)phenol metabolites to passively cross the BBB according to different *in silico* models are reported in Table 2. The number of studies found on LMW (poly)phenol metabolites passive permeation was quite limited and data was only found for 23 of the described molecules in Table 1. Overall, most of the molecules evaluated demonstrate some probability of crossing the BBB, although this probability cannot be quantitative, it is indicating only that if the presence is expected in the brain it is due to passive permeation. One of the exceptions is salicylic acid where *in silico* predictions projected as not probable to reach the brain. However, this molecule has been detected in the rat brain and its presence was associated with transport-mediated mechanisms [36]. For some of these compounds like protocatechuic and, vanillic acid different estimates were given by different predictive models, demonstrating that the model used can influence the conclusions taken about passive permeability. All molecules analyzed in QikProp software seemed to indicate, through log BB, that the molecules could reach the brain. Nevertheless, different conclusions were drawn with SWISS ADME where di and tri-substituted phenolic acid metabolites (vanillic acid, protocatechuic acid and, gallic acid) were stated as not probable of crossing the BBB. Furthermore, SWISS ADME predicts ferulic acid has a molecule able to cross the BBB, together with p-coumaric and cinnamic acid but not sinapic acid. Information regarding other classes of LMW (poly)phenol metabolites like hippuric acids and propionic acids would greatly increase our knowledge of passive permeation for these types of molecules. Nevertheless, this data is still missing. Overall, *in silico* predictions since are less labor-intensive, inexpensive and high throughput, are quite useful for initial information about the potential of a metabolite to cross the BBB. Initial *in silico* screening in a set of compounds allows refining further analysis in the more technically challenging process of obtaining detailed *in vivo* BBB permeability data for the most promising compounds. A good example of this rationale was the comprehensive study that was done for the flavan-3-ols metabolites, phenyl-*γ*-valerolactones, phenylvaleric acids, and their conjugates. This study explored through an *in silico* analysis the most promising molecules which were tested in a cell-based assay. Finally, the presence of the above mentioned compounds was validated in the brain in three different *in vivo* studies [33]. It will be very interesting to perform a comparative study for all described LMW (poly)phenols metabolites in the same model of passive permeation - an evaluation that, to the best of our knowledge, to date still has not been done.

**Table 2:** – *In silico* studies on LMW (poly)phenol metabolites for passive permeation across the BBB

Class	Common denomination	Modelling^1^	BBB predicted permeation	References
Benzene diol and triols	Catechol	PreADMET v2.0	Probable	[37]
	catechol- *O*-sulfate	QikProp 2015-4	Probable	[25]
	pyrogallol- *O*-sulfate	QikProp 2015-4	Probable	[25]
	1- *O*-methylpyrogallol- *O*-sulfate	QikProp 2015-4	Probable	[25]
	2- *O*-methylpyrogallol-1- *O*-sulfate	QikProp 2015-4	Probable	[25]
	4-methylcatechol- *O*-sulfate	QikProp 2015-4	Probable	[25]
Benzaldehydes	Protocatechualdehyde	QikProp 2009	Probable	[38]
	benzoic acid	StarDrop	Probable	[39]
		PreADMET v2.0	Probable	[37]
	salicylic acid	*In house based on GROMACS 4.5.5*	Not probable	[40]
	4-hydroxybenzoic acid	SWISS ADME	Probable	[41]
		PreADMET v2.0	Probable	[37]
	protocatechuic acid	QikProp 2009	Probable	[38]
		SWISS ADME	Not probable	[41]
		PreADMET v2.0	Probable	[37]
Benzoic acids	vanillic acid	QikProp 2009	Probable	[38]
		SWISS ADME	Not probable	[41]
		PreADMET v2.0	Probable	[37]
	vanillic acid-4- *O*-sulfate	QikProp 2015-4	Probable	[25]
	gallic acid	SWISS ADME	Not probable	[41]
	4-*O*-methylgallic acid	QikProp 2015-4	Probable	[25]
	4-*O*-methylgallic acid-3-*O*-sulfate	QikProp 2015-4	Probable	[25]
	syringic acid	SWISS ADME	Not probable	[41]
Phenylacetic acids	2-(4-hydroxyphenyl)acetic acid	PreADMET v2.0	Probable	[37]
	caffeic acid	QikProp 2009	Probable	[38]
	ferulic acid	QikProp 2009	Probable	[38]
		SWISS ADME	Probable	[41]
Cinnamic acids	*p*-coumaric acid	SWISS ADME	Probable	[41]
	sinapic acid	SWISS ADME	Not probable	[41]
	cinnamic acid	SWISS ADME	Probable	[41]

^1^The probability of the molecules to passively cross the BBB is defined by each of the models used: QikProp: Molecules probable of crossing the BBB should have a log BB between -3 and 1.2 [42]. StarDrop: Molecules are classified by the authors as not probable or probable according to the log BB of -1 or higher than -1 respectively [39]. *In house* model using GROMACS 4.5.5: Molecules are probable of crossing the BBB if logP_eff_ is above 0 [40]. SWISS ADME: Based on the information given by the authors, molecules are probable to reach the brain if Log P_o/w_ is in the range of 1.5 to 2.7 [41]. PreADMET v2.0: Authors present BBB crossing probability as a percentage. All molecules with BBB(%) above 0.1 are considerately probable of crossing the BBB [37].

### Cell based data on BBB permeability

*In vitro* cell-based studies, mostly taking advantage of brain endothelial cellular models, have been a very important tool to address the ability of metabolites permeation across the BBB endothelium in a more realistic way. For some flavonoids and their known circulating metabolites, the permeability was tested on *in vitro* models of the BBB [21]. The uptake of hesperetin, naringenin, and their respective *in vivo* glucuronides, as well as the anthocyanins cyanidin-3-rutinoside and pelargonidin-3-glucoside has been reported in two brain endothelial cell lines from mouse (b.END5) and rat (RBE-4). In this study flavonoids permeation across *in vitro* BBB mono layers was compared to the potential for permeation for their more polar glucuronidated conjugates and to specific phenolic acids derived from colonic biotransformation of flavonoids. The authors concluded that hesperetin and naringenin present a high apparent permeability [21]. Moreover, in another study, both catechin and epicatechin were able to cross two BBB cell lines, RBE-4 cells and hCMEC/D3 (immortalized human cerebral micro vessel endothelial cell line) in a time-dependent manner [43]. *In vitro* transmembrane transport of flavonols, flavan-3-ols and anthocyanins and some of their methylated and glucuronidated metabolites were also observed in the hCMEC/D3 cells [44]. Interestingly, the authors concluded that in most cases, the metabolites exhibited higher transport efficiency than their unconjugated parent compounds [44]. Overall, data strongly suggest the effective uptake of dietary (poly)phenols and their metabolites through the BBB endothelium. However, there are very few studies focusing on the LMW (poly)phenols metabolites and their ability to reach the brain. In fact, a huge potential in this field has been underestimated, and only for a few classes such as benzene diols and triols, benzoic acid, and cinnamic acid data was collected (Table 3).

**Table 3:** – Evidence of transport of LMW (poly)phenol metabolites in cellular models of the blood-brain barrier based in brain endothelial cells

Compound class	Common name	Cell model	Concentration (*μ*M)	Time (h)	Transport (%)	References
Benzene diols and triols	catechol-*O*-sulfate	HBMEC	5	2	7.7±0.6	[25]
	pyrogallol-*O*-sulfate				4.5±0.1
	1-O-methylpyrogallol-*O*-sulfate				1.3±0.4
	2-O-methylpyrogallol-1-*O*-sulfate				4.2±0.4
	4-methylcatechol-*O*-sulfate				12.6±1.9
	pyrogallol	BBB kit (RBT-24)^1^	30	0.5	2.84±0.27	[45]
	pyrogallol-1-*O*-glucuronide	1.91±0.29
Benzoic acids	vanillic acid-4-O-sulfate	HBMEC	5	2	2.5±1.2	[25]
	4- *O*-methylgallic acid	4.4±2.1
	4- *O*-methylgallic acid-3-*O*-sulfate	7.9±0.7
	gallic acid	BBB kit (RBT-24)^1^	30	0.5	6.5±0.6	[46]
Cinnamic acids	ferulic acid	BBB kit (RTU)^2^	10	1	medium	[47]
					capacity^3^
Phenylpropionic acids	dihydroferulic acid	BBB kit (RTU)^2^	10	1	medium	[47]
	dihydrocaffeic acid				capacity^3^

Abbreviations: HBMEC, Human Brain Micro vascular Endothelial cells; BBB, Blood-brain barrier. ^1^BBB Kit™ (RBT-24) is an*in vitro* model of BBB made of primary cultures of rat (Wistar rat) brain capillary endothelial cells, brain pericytes, and astrocytes. ^2^BBB kit (RTU) is an *in vitro* model of BBB consisting of endothelial cells from bovine brain capillaries. ^3^According to the authors, compounds were classified into 3 categories considering their ability to cross the BBB, based on their permeability coefficient (Pe): low capacity (Pe < 1×10^–3^ cm.min^–1^), medium capacity (1×10^–3^ cm.min^–1^ ^<^ Pe < 2×10^–3^ cm.min^–1^) or high capacity (Pe > 2×10^–3^ cm.min^–1^). Pe values were calculated for ferulic acid (1.31±0.20 Pe (×10^–3^ cm.min^–1^), dihydroferulic acid 1.20±0.24 Pe (×10^–3^ cm.min^–1^) and dihydrocaffeic acid 1.01±0.1 Pe (×10^–3^ cm.min^–1^).

In a recent study, the endothelium transport of some known LMW (poly)phenol metabolites, products of sulfate conjugations was addressed by using a human brain micro vascular endothelial cell (HBMEC) line [25]. The same model was used to confirm flavan-3-ols metabolites BBB permeability followed by *in vivo* validation [33]. The BBB cellular models consist of the ability to cultivate cells on a transwell that divides two separate chambers mimicking the luminal (blood) and the abluminal (brain) sides separated by brain micro vascular endothelial cells. Catechol-*O*-sulfate, pyrogallol-2-*O*-sulfate, 1-*O*-methylpyrogallol-*O*-sulfate, 2-*O*-methylpyrogallol-1-*O*-sulfate, vanillic acid-4-*O*-sulfate, 4-*O*-methylgallic acid, and 4-*O*-methylgallic acid-3-*O*-sulfatehave been tested for BBB permeability and all of them were detected in the abluminal side [25]. However limited amounts of these LMW metabolites were observed to be transported, ranging from 1.3 to 12.6% of transport [25]. One of the possibilities to originate the low passive diffusion could be the involvement of efflux transporters pumping back the metabolites to the luminal side. In the same study the efflux transporters P-gp, BRCP, and MRP1 activity were tested in the cells for the transport of some LMW (poly)phenols metabolites and the percentage of transport was not significantly affected [25]. Interestingly, phase II derivatives, sulfated and methylated, presented a higher percentage of transport comparing to their counterparts, suggesting being more prone to be brain permeable. In any case, passive permeation may not be the only method of transport across the BBB, since it could also be mediated by carriers [25].

Some ready-to-use BBB model kits were already tested for LMW (poly)phenol metabolites BBB permeability. In that sense, resorting to the BBB kit (RBT-24), made of primary cultures of rat brain endothelial cells, pericytes and astrocytes, the transport for gallic acid, pyrogallol and pyrogallol-1-O-glucuronoide was demonstrated [45,46]. Despite of the pyrogallol is less polar than gallic acid, this last one presented a higher permeation since it is known that it can be incorporated into cells through an organic anion carrier (OAT3) [48]. Furthermore, another commercially available *in vitro* model of BBB (RTU), consisting of bovine endothelial cells, was used to test the potential of ferulic acid, dihydroferulic acid, and dihydrocaffeic acid to cross the BBB [47]. In fact, none of these metabolites presented a high capacity to cross these endothelial cells, presenting permeability coefficient (Pe) values slightly above the adopted cutoff value of 1×10^–3^ cm.min^–1^ (Table 3). This means that their permeation to the brain could be considered as negligible. This medium capacity to cross the BBB, could result, as mentioned before, from a low passive diffusion or even can be due to the involvement of efflux transporters pumping back the metabolites to the luminal side. In opposition, in Madin-Darby Canine Kidney (MDCK) cells, which is considered a useful model to evaluate cell permeability, ferulic acid rapidly crossed this barrier, increasing the transport rate overtime, reaching approximately 25% [49]. In a different BBB model consisting of ECV-304 bladder carcinoma cells and C6 glioma cells in co-culture, also described as a good indicator of BBB permeability, the authors were unable to detect the transport of (3,4-dihydroxyphenyl) acetic acid, 3-(4-hydroxyphenyl) propionic acid and 2-(4-hydroxyphenyl) acetic acid [21].

Interestingly, *in silico* predictions about the permeability of the specific molecules with available information show corroborating data between *in vitro* and *in silico* predictions. However, the number of *in vitro* studies is still limited to generalize this validation of the *in silico* data.

Still, although the data available regarding LMW (poly)phenols BBB permeability is scarce, it has gained a lot of attention since the BBB represents the entrance door to reach the brain. Noteworthy, sometimes the goal of the research is to find methods to avoid brain penetration of toxins and potentially dangerous molecules but sometimes it is convenient to promote the entrance of drugs. An interesting branch of research has been focusing on increasing the efficiency of drug delivery with the purpose of overcoming the tight regulation characteristic of the BBB. In that sense, sinapic acid, conjugated with a zwitterionic polymer in an encapsulated bovine serum albumin-based nanoparticles, proved to be a novel bioinspired BBB-permeable ligand for delivery of cargo into the brain [50], which is seen as a key factor for the therapeutic efficacy in the treatments of CNS-related diseases.

### In vivo experiments confirm the presence of LMW (poly)phenol metabolites in the brain

For a full comprehension regarding the brain uptake of phenolic metabolites, *in vivo* experiments are crucial since they are the only way to fully recapitulate the complexity of the BBB. Being this a very emergent issue, several studies have been interested in confirming the brain bioavailability of (poly)phenols, having already been identified some of them in different brain regions of rats [51, 52] and pigs [53, 54] which can accumulate in a non regional-specific manner [55]. Micro dialysis sampling in rats also showed the presence of (+)-catechin and (–)-epicatechin in the brain, demonstrating their ability to cross the BBB [56]. The exact localization and target sites of (poly)phenols in the brain represent an important aspect for understanding its molecular mechanism but this issue remains unlooked. The main reason for this is the fact that the major analytical approach for this evaluation in biological samples relies on chromatographic techniques. Some attempts to address this was done by using monoclonal antibodies for the compounds [57, 58]. Also, the brain presence of (poly)phenols seems to be independent of their route of administration, since different compounds were detected in the brain after different types of administration. For instance, epigallocatechin gallate [59, 60], epicatechin [61–64], anthocyanins [52, 65], quercetin [66, 67] and naringenin [68], were detected in the brain after oral administration while naringenin and its glucuronide were also detected after intravenous injection [69] as well as hesperetin that was detected in the brain striatum [70]. However, there are some contradictory results as is the case of anthocyanins that could not be detected in brains of rats after supplementation with raspberry juice in a dose equivalent to 700 mL to a 70 kg human [71] but were detected in other experimental designs that involved either higher doses or chronic administration [52, 72, 73]. For analysis of a compound penetration in the brain it is the gold standard to use exsanguinated or perfused animals or a correction for residual blood in the brain [23]. This was one critical aspect that has raise some controversy since these appropriated control procedures were lacking in some studies.

By contrast to the dietary (poly)phenols, only a very limited number of studies have focused on LMW (poly)phenol metabolites, although some evidence can be found of their potential to cross the BBB and reach the brain. Among predictive studies, whose outcomes provide an idea about the ability of metabolites to cross the BBB endothelium by passive diffusion and *in vitro* studies that can give us a wide-ranging clue about their permeation, both approaches are still limited. On the other hand, *in vivo* models, despite being based on rodent and pig models, are those that get closest to recapitulate the human physiology, undergoing the influence of natural processes of a living organism. This means that in animal studies the brain bioavailability of phenolic metabolites will be influenced by different cell types, enzymes, transporters, endogenous molecules, etc. mimicking in a better way the organism environment.

Although limited, evidence of the BBB capacity to uptake LMW (poly)phenol metabolites using *in vivo* models have been increasing in the literature. However the main findings attained in these studies, have different experimental designs, particularly regarding the animal model, the gender, the type of administration, the dose, and the compounds sourced, hampers an exact comparison. Within this section, we review some of these studies, highlighting the compounds that were tested to cross the BBB, the dosage, and the brain uptake, using *in vivo* models. An important difference in these studies, that lead to different conclusions, is the type of administration of the (poly)phenols. Compounds were sourced either by extract form or pills being administered intragastrically or orally mixed with the diet (Table 4), or they can be administered themselves directly either by intravenous or intraperitoneal injection (Table 5). The difference between these two approaches is the metabolic route that compounds will follow. If they enter the organism orally, the pharmacokinetics of the metabolites increase the difficulty for the exact quantification of the amount that reaches the brain and the calculation of the proportion that is brain-bioavailable. The extensive metabolism over the (poly)phenols, present in the extracts, by the microbiota and the entero-hepatic enzymes, will lead to difficulties in estimating the concentration of a specific metabolite in blood and the kinetics (T_max_, T_1/2_) associated with their presence. On the other hand, if the administration is directly in the blood stream it will bypass most hepatic metabolic pathways, reaching the tissues faster and making it easier to know with more accuracy the percentage that reached the brain.

**Table 4:** – Evidence of *in vivo* brain uptake of LMW (poly)phenol metabolites after oral administration

Compound class	Common name	Study design								Brain uptake	References
		Animal model	Food Matrix	Dosage	Units	Frequency	Type of administration	Euthanasia method	Time collection of brain after uptake	(nmol/g of tissue)
Benzoic Acids	benzoic acid	Wistar rats (male)	GSPE^1^	125	mg/Kg BW	Single	Intragastric	Exsanguination	2 h	15.63±2.40	[74]
				250						2.53±1.40
				375						18.88±2.96
				1000						11.00±3.95
		Wistar rats (male)	GSPE^1^	100	mg/Kg BW	Repeated (once daily for 12 weeks)	Oral	Exsanguination	21 h	1.466±0.289	[75]
	3-hydroxybenzoic acid	Wistar rats (male)	GSPE^1^	125	mg/Kg BW	Single	Intragastric	Exsanguination	2 h	0.61±0.13	[74]
				250						0.43±0.18
				375						0.44±0.04
				1000						1.06±0.06
		Wistar rats (male)	GSPE^1^	100	mg/Kg BW	Repeated (once daily for 12 weeks)	Oral	Exsanguination	21 h	0.635±0.081	[75]
		Spague-Dawley rats (male)	GSPE^2^	25	mg/Kg BW	Repeated (once daily for 11 days)	Intragastric	Perfusion	6 h	1.36±0.14	[76]
				250						1.75±0.30
	protocatechuic acid	Balb/cA mice	Powder diet	2 g of PCA mixed with 98 g of diet	g	Supplied for 12 weeks	Oral	Not described	0.26±0.10	[77]
				4 g of PCA mixed with 96 g of diet						0.41±0.14
		Spague-Dawley rats (male)	Danshen extract^3^	5	mL/Kg BW	Single	Intragastric	Perfusion	∼ 0.6±0.5 ^7^	[78]
	gallic acid	Sprague-Dawley rats (male)	GSPE^4^	50, 100 and 150	mg/Kg BW	Repeated (dose-escalation for 10 days)	Intragastric	Perfusion	Detected (trace levels)	[79]
	vanilic acid	Wistar rats (male)	phenolic extract from olive cake	3	g/Kg BW	Single	Intragastric	Exsanguination	1 h	1.8	[80]
									2 h	1.7
									4 h	2.0
Phenylacetic acids	homovanillic acid	Wistar rats (male)	GSPE^1^	125	mg/Kg BW	Single	Intragastric	Exsanguination	2 h	2.36±0.48	[74]
				250						2.11±0.23
				375						1.84±0.44
				1000						2.39±0.40
		Wistar rats (male)	GSPE^1^	100	mg/Kg BW	Repeated (once daily for 12 weeks)	Oral	Exsanguination	21 h	0.528±0.072	[75]
		Sprague-Dawley (male and female)	Hydroxytyrosol^5^ (Seprox Biotech, Madrid, Spain)	1	mg/Kg BW	Single	Intragastric	Not described	5 h	0.39±0.04^8^	[81]
				10						0.32±0.02^8^
				100						0.40±0.05^8^
Hippuric acids	hippuric acid	Wistar rats (male)	GSPE^1^	125	mg/Kg BW	Single	Intragastric	Exsanguination	2 h	0.32±0.10	[74]
				250						0.56±0.18
				375						0.42±0.21
				1000						0.22±0.01
Cinnamic acids	ferulic acid	Spague-Dawley rats (male)	*Shunaoxin* pills	20 pills ground into powder	Single	Intragastric	Not described	2^9^	[49]
Phenylpropionic acids	3-hydroxyhydrocinnamic acid	Spague-Dawley rats (male)	GSPE^2^	25	mg/Kg BW	Repeated (once daily for 11 days)	Intragastric	Perfusion	6 h	1.49±0.14	[76]
				250						2.53±0.68
	4-hydroxyhydrocinnamic acid	Wistar rats (male)	Hazelnut extract^6^	5	g/Kg BW	Single	Intragastric	Exsanguination	2 h	15±01.1	[82]

Abbreviations: GSPE,Grape seed (Poly)phenol extract; PCA, Protocatechuic acid; BW, Body weight. ^1^GSPE was provided by used was Meganatural-AZ^®^ GSPE as described by the authors. ^5^Hydroxytyrosol was provided by Seprox Biotech (Madrid, Spain)as described by the authors. ^6^Hazelnut extract was provided by La Morella Nuts S.A. (Reus, Spain) as described by the authors was a source of procyanidins. ^7^The original value (0.09±0.07*μ*g of protocatechuic acid /mL of microdialysis brain sample) presented by the authors was converted to nmol/g of tissue considering the brain density of 1 g.cm^-3^ and the molecular weight of PCA (154.12 g/mol). ^8^Homovanillic acid was also detected in the vehicle group and only for the highest dose (HT-100) homovanillic presented significant increases in the brain. ^9^The original value (0.4*μ*g of ferulic acid/g of wet tissue) presented by the authors was converted to nmol/g of tissue considering the molecular weight of ferulic acid (194.18 g/mol).

**Table 5:** – Evidence of *in vivo* brain uptake of LMW (poly)phenol metabolites after either intravenous or intraperitoneal injection of the metabolites

Compound class	Common name	Study Design					Brain uptake	References
		Animal model	Dosage	Units	Type of administration	Euthanasia method	(nmol/g of tissue)
Benzene diols and triols	catechol	Inbred strain of mice (male)	5.5×10^5a^	nmol/Kg BW	Intraperitoneal	Decapitation	290^d^	[83]
		Inbred strain of mice (female)	5.5×10^5a^			Decapitation	304^e^	[84]
	pyrogallol	Inbred strain of mice (male)	1.1×10^6b^			Decapitation	350^f^	[83]
		Inbred strain of mice (female)	4.8×10^5c^			Decapitation	220^g^	[84]
Benzoic acids	vanillic acid	Wistar rats (male)	50	nmol	Intravenous	0.385	[51]
	4-hydroxybenzoic acid		100			Decapitation^h^	0.408
	gallic acid		900				0.611
Phenylacetic acids	homoprotocatechuic acid		75				0.501
	homovanillic acid		50				0.433
Hippuric acids	4-hydroxyhippuric acid		250				0.020
Cinnamic acids	caffeic acid		60				0.0038
	ferulic acid		60				0.027
Phenylpropionic acids	3-hydroxyhydrocinnamic acid		450				0.193

Abbreviations: BW,Body weight. ^a^The original value (60 mg/Kg BW) presented by the authors was converted to nmol/Kg BW considering the molecular weight of catechol (110.1 g/mol). ^b^The original value (120 mg/Kg BW) presented by the authors was converted to nmol/Kg BW considering the molecular weight of pyrogallol (126.1 g/mol). ^c^The original value (60 mg/Kg BW) presented by the authors was converted to nmol/Kg BW considering the molecular weight of pyrogallol (126.1 g/mol). ^d^The original value (32*μ*g/g of tissue) presented by the authors was converted to nmol/g of tissue considering the brain density of 1 g.cm^–3^ and the molecular weight of catechol (110.1 g/mol). ^e^The original value (33.5*μ*g/g of tissue) presented by the authors was converted to nmol/g of tissue considering the brain density of 1 g.cm^–3^ and the molecular weight of catechol (110.1 g/mol). ^f^The original value (44*μ*g/g of tissue) presented by the authors was converted to nmol/g of tissue considering the brain density of 1 g.cm^–3^ and the molecular weight of pyrogallol (126.1 g/mol). ^g^The original value (28.4*μ*g/g of tissue) presented by the authors was converted to nmol/g of tissue considering the brain density of 1 g.cm^–3^ and the molecular weight of pyrogallol (126.1 g/mol). ^h^As the concentrations of the metabolites in the brains may be significantly influenced by the amount of each metabolite present in the residual blood, the authors have made a correction by estimating the amount of each metabolite in intravascular blood present in the brain, considering the volume of brain blood as 47.7*μ*L/g.

Margalef et al. have administered orally different acute doses (125, 250, 375, and 1000 mg/Kg BW) of grape seed (poly)phenol extract (GSPE) to the male rats [74]. The total of 16 different phenolic acids that generate a set of phase II metabolites found at different concentrations in different tissues were identified from this extract [74]. As expected, given that the brain is a peripheral organ difficult to cross, metabolites that were quantified in this tissue presented much lower concentrations than those found in the liver and kidney. Notably, 4 of the human LMW (poly)phenol metabolites (listed on Table 1) were detected in brain plus phenylpropionic acid, an LMW (poly)phenol metabolite detected in rodents, being that a proportional increase with the increased dose of GSPE was not observed. Benzoic acid, 3-hydroxybenzoic acid, homovanillic acid, and hippuric acid were detected after 2 hours of GSPE ingestion. In turn, gallic acid, 3-O-methylgallicacid, and 4-hydroxyhydrocinnamic acid were not detected in the brain of these rats for any dose tested, despite being detected in other organs in the same animals. Moreover, gallic acid was present in the extract and 4-hydroxyhydrocinnamic acid could be generated by the metabolism of quercetin-3-O-galactoside, which was also present in GSPE [74]. The same authors evaluated if the GSPE extract could originate a brain accumulation of the metabolites after a long-term administration in the rats [75]. The authors concluded that the concentrations detected in the brain, 21 hours after daily GSPE administration of 100 mg/Kg BW for 12 weeks, do not reflect an accumulation but instead originated from the last acute administration dosage [75]. This conclusion was based on the fact that flavanol metabolites in plasma were not detected 21 hours after GSPE administration [75]. Moreover, very few metabolites were detected in the studied tissues and at levels much lower than the total amounts that were quantified 2 hours after a single GSPE ingestion of a similar dose in the previous study [74]. In the study, conducted by Wang et al., also using GSPE administered for 11 days, the content for 12 LMW (poly)phenol metabolites in the brain were analyzed [76]. Among the 12 screened compounds 8 were found in the brain but only 3-hydroxyhydrocinnamic acid and 3-hydroxybenzoic acid were found significantly increased compared with the control animals (4.5 and 2.5-fold change respectively). Hippuric acid, 4-hydroxybenzoic acid, 3-hydroxyphenylacetic acid, homoprotocatechuic acid, dihydrocaffeic acid and phenylacetic acid were also detected in the brain, but no differences were found compared with rats without GSPE [76]. This differential profile of the LMW (poly)phenol metabolites detected in the brain using the same source the GSPE extract could be due to several reasons. One possibility is the time of analysis, in the latter study, the analysis of LMW (poly)phenol metabolites present in the brain was done 6 hours post-GSPE administration [76] the opposite of 2 and 21 hours post-administration in the previous studies [74, 75]. LMW (poly)phenol metabolites appearance in circulation *in vivo* peaks around 6 hours post-consumption and have longer elimination half-life [85, 86]. Accordingly, it is not clear if a chronic consumption *vs.* acute ingestion could impact the levels detected since there is no a direct comparison for the same time point of tissue collection after the last intake. Another aspect that may account for different results is the fact that these studies have used different rat strains (Sprague-Dawley *vs.* Wistar rats) whose endocrine system was described as different and this causes differences in food conversion and food intake [87]. Altogether, these differences in the experimental design may justify the qualitative and quantitative differences found in the rats’ brains of LMW (poly)phenol metabolites in both studies.

Concerning the presence of gallic acid in the brain, another study also using Sprague-Dawley rats, likewise has not detected this metabolite in the brain when rats were orally gavaged with acute doses of GSPE (50, 100 or 150 mg/Kg BW) [79]. Only upon repetitive dosing with GSPE for 10 days, trace levels of gallic acid were detected [79]. In another study, gallic acid was also not found in the brain of control animals that were orally treated with Gualou Guizhi granules, a standard prescribed drug from Traditional Chinese Medicine. In the same trial, however, the rats that were subjected to cerebral ischemia/reperfusion injury and, treated also with the Gualou Guizhi granules, presented higher permeability of BBB, favouring the passage of several metabolites, including the gallic acid [88]. The BBB integrity is an important factor, even though BBB is highly regulated, towards an insult or a disease it may be disrupted, and therefore it increases its permeability, favouring the entrance of molecules that otherwise would not enter.

Protocatechuic acid was detected in the brain of rats that received Danshen extract intragastrically (containing danshensu 40 mg/Kg BW, protocatechuic aldehyde 149 mg/Kg BW and salvianolic acid B 50 mg/Kg BW) [78]. Additionally, male Balb/cA mice with a diet supplemented with protocatechuic acid have also shown increased levels of this metabolite in the brain, which were not detectable in brains from mice not supplemented [77]. Ferulic acid was also found in the brain of rats that took Shunaoxin pills, a traditional Chinese medicine product enriched in ferulic acid. It was showed that ferulic acid is rapidly absorbed and distributed in the brain, being detectable 5 minutes after the administration and no longer at 4 hours [49].

Although not derived from colonic metabolism, metabolites derived from intake of hydroxytyrosol food sources, like virgin olive oil, are also LMW (poly)phenol metabolites. Homovanillic acid and some related metabolites detected in rodents like homovanillic acid sulfate and homovanillic alcohol sulfate, were detected in the brains of the rats 5 hours after an acute high dose of hydroxytyrosol (100 mg/Kg BW) [81]. All these metabolites appear in the brain of control animals derived from dopamine metabolism which masks the appearance of the metabolites for the intake of lower doses of hydroxytyrosol. Homovanillic alcohol sulfate was also reported to increase in brains of rats that consumed daily standard diet supplemented with 5 mg/Kg BW of either hydroxytyrosol or hydroxytyrosol derivatives for 21 days [89]. Moreover, sulfates of vanillin, vanillic acid and homovanilic were also detected in the brain of rats after the ingestion of phenolic extract from olive cake [80].

Interestingly a significant gender effect in the plasma, liver, and kidney concentrations of hydroxytyrosol metabolites was observed [81]. However, no gender effect was observed in the uptake of hydroxytyrosol metabolites in the brain and heart [81]. On the other hand, gender effects were considered to influence brain uptake of (poly)phenols in the case of GSPE derived flavanol metabolites where differences between males and females were observed [74, 90, 91]. Being (poly)phenols recognized by the human organism as xenobiotics, it is not surprising to observe differences between genders in its metabolism. Such differences can be potentiated by age, estrous/menstrual cycle and pregnancy. Generally, it is described that male rodents tend to have higher levels of cytochrome P450, while phase II metabolism seems to be quite similar between genders. Some reports, however, show higher glucuronidation in males in the liver, compensated with higher glucuronidation in the female kidney. Moreover, some CYP450 expression seems to be sex-specific, and their expression levels quite different between genders meaning the rate of metabolism and excretion can be highly dependent on the substrate [92]. Gender affects the balance between the sulfation, glucuronidation, and methylation of (poly)phenols and the way how xenobiotics affect metabolic enzymes in the liver of rats is also different between sexes [90]. It has been reported for *in vitro* models of BBB that estrogens may play a role in modulating free flavanol uptake, and it has been further suggested that progesterone can act as an endogenous factor that modulates P-glycoprotein ability to serve as transporters of flavan-3-ol’s across the BBB [43].

Another approach to evaluate brain permeability to (poly)phenol metabolites *in vivo* is its direct administration to the circulation bypassing the gastrointestinal tract (Table 5). In that sense, a pharmacokinetic study tried to uncover the fate of microbial metabolites of dietary (poly)phenols upon intravenous injection in the dorsal penis vein of rats [51]. The injection site allows the minimal manipulation of anesthetized animals avoiding the release of inflammatory mediators that might affect BBB permeability, the normal distribution, and excretion of metabolites. From the 23 metabolites present in the mixture injected, 18 are LMW metabolites described in Table 1. From these, 13 were found in control brains as endogenous but only 9 were found increased compared to the control group (Table 5). Among them, caffeic acid has reached the brain at 15 minutes at approximately 34 times the basal concentration (0.11 pmol/g of tissue) and has presented the biggest difference between controls and injected rats, but it was the one detected in lower concentration (3.81 pmol/g of tissue). On the other hand, vanillic acid presented the highest percentage of transport to the brain considering both the dose injected and the mean concentration found in control brains [51]. Interestingly within the 9 LMW (poly)phenol metabolites, the top 4 that presents the higher percentage of transport are all phenolic acids (gallic acid < 4-hydroxybenzoic acid < homoprotocatechuic acid < homovanillic acid < vanilic acid). In fact, an interesting consideration could be made about these molecules, since homovanillic acid, one of such top 4 molecules was already described to be transported in rats brain by the activity of the Organic Anion Transporter 3 (rOAT3) [93]. Based on this, we may expect that this type of transport could also be occurring for other phenolic acids.

In an attempt to identify circulating hydroxytyrosol metabolites and their deposition in tissues, D'Angelo, and colleagues injected intravenously radiolabelled ^14^C hydroxytyrosol in Sprague-Dawley rats [94]. Although the radioactivity up taken by the brain was lower than in other organs, besides the hydroxytyrosol, homovanillic alcohol, 3,4-dihydroxyphenylacetaldehyde, 3,4-dihydroxyphenylacetic acid, homovanillic acid and sulfate conjugates were also detected in the brain. The authors consider that some of these metabolites were presumably derived from enzymatic activities operating in the brain, like methylation that reflects COMT activity [94]. In fact, in order to evaluate the effects of hydroxytyrosol and its nitro derivatives on COMT activity, compounds were administered into the rat striatum through a microdialysis probe [95]. All compounds increased extracellular levels of 3,4-dihydroxyphenylacetic acid during the perfusion and did not produce a decrease in the extracellular homovanilic acid suggesting their effect as COMT inhibitors [95]. The same results were observed in rats that received either an acute (single dose; 20 mg/Kg, i.p.) or chronic (one daily dose for 5 days; 20 mg/Kg, i.p.) treatment with the compounds [96].

Another study has addressed the penetration of catechol and pyrogallol into male mouse brain after intraperitoneal injection of 60 mg/Kg BW and 120 mg/Kg BW respectively, being both compounds detected in the brain [83]. Similar observations were made in female mice after the administration of both compounds intraperitoneally [97]. In both studies, catechol and pyrogallol reached the brain at very high concentrations, reflecting the supraphysiological concentrations administered to mice. Although it was not the goal of these studies, the results do not indicate noticeable sex differences in the ability of the compounds to reach the brain. However, this could be an important factor to be considered in futures studies, since the sex dimorphisms in many aspects of COMT’s function are already described and therefore relevant for benzene diols/triols and also dihydroxyphenylacetic acids. For instance, it was detected a prominent sex differences in the impact of tolcapone, COMT inhibitor, on dopamine metabolite levels. Specifically, females showed a greater tolcapone-related change in 3,4-dihydroxyphenylacetic acid in prefrontal cortex and cerebellum, and in homovanilic acid in the prefrontal cortex and striatum [98]. In fact, gender effects were already considered to influence (poly)phenol brain uptake as previously discussed.

Concerning these data, different conclusions can be drawn if the proper interpretation is not made. There are many variables between studies regarding their experimental designs that influence the amount and type of LMW (poly)phenol metabolites detected in the brains that should be taken into consideration. From the studies herein presented, the source of (poly)phenols is quite wide and variable and even the regular diet given to the rats can be different. The time of fasting and the time after the last (poly)phenol administration and the quantification are critical parameters that generate different results. As we already highlighted, the rat strain and gender, the concentration of the compound, as well as the route and the frequency of the administration have also a strong influence on the amount and type of LMW metabolites able to reach the brain.

## BBB TRANSPORTERS AND FURTHER METABOLISM

**Fig. 1:**
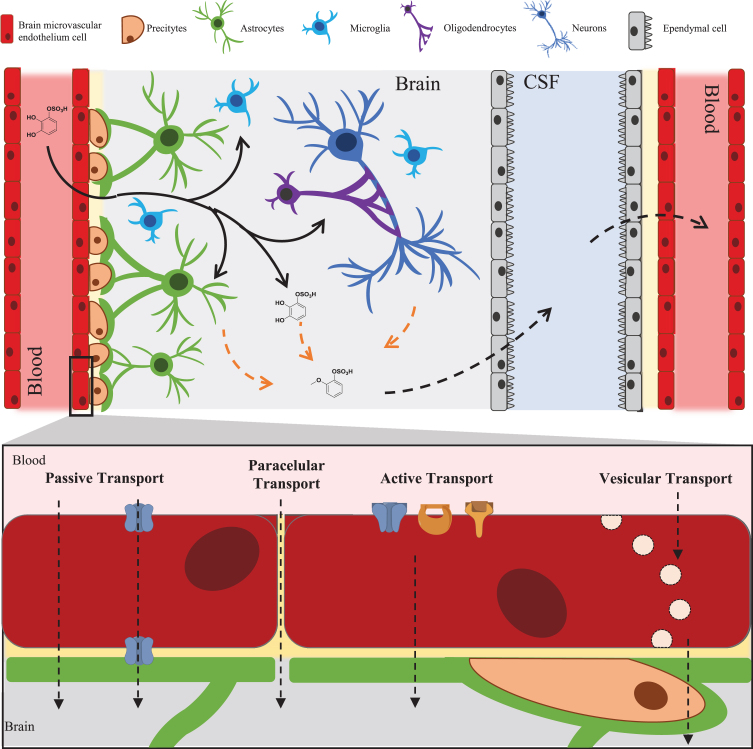
– LMW (poly)phenol metabolites journey to and through the brain. Black arrows represent passage and distribution of the molecules into the brain. Dashed orange arrows represent putative metabolism inside the brain. Dashed arrows represent possible methods of distribution to and from the brain observed for other xenobiotics and predicted for LMW (poly)phenols metabolites.

The mechanisms by which LMW (poly)phenol metabolites cross the BBB are still quite underexplored. As stated previously, passive permeation and carrier mediated transport could constitute a potential mechanism by which these molecules could cross the BBB and potentially reach the brain. However, other mechanisms of transport are also considered, such as paracellular transport and vesicular transport (Fig. 1). Studies with parent flavonoids suggest that they are transported by ABC, OAT and also OATP [28, 29] transporters and that are responsible for most of the absorption and distribution of flavonoids within the body as well as their excretion in urine [3]. On the other hand several ABC transporters were identified in the BBB such as P-pg (ABCB1), MRP1 (ABCC1), MRP2 (ABCC2), MRP4 (ABCC4) and BCRP (ABCG2) [99]. Therefore, it is plausible to consider that the same transporters could be involved in the transport of the residual levels of flavonoid circulating and most importantly the transport of their more abundant LMW (poly)phenol metabolites. Yet, a study using HBMEC cells showed no impact of P-gp, MRP1 or BCRP on the transport of some of these metabolites [25]. For some LMW (poly)phenol metabolites like phenolic acids, interactions with OAT transporters such as OAT1 (SLC22A6), OAT3 (SLC22A8), and OAT4 (SLC22A11) was already demonstrated [100, 101]. The transport of 3,4-dihydroxyphenylacetic acid and homovanilic acid, two LMW (poly)phenol metabolites but also products of dopamine metabolism (Table 1) are known to be transported across the BBB through OAT3 [93, 102]. This observation could reveal an important starting point for understanding how these molecules traverse the brain and reach the cerebrospinal fluid [93, 102]. Glucuronidation could also be a way of transport for xenobiotics. Glucuronide metabolites of morphine have been suggested to be transported across the BBB by glucose transporter 1 (GLUT1), organic anion transporters (OATs) and organic anion-transporting polypeptide (OATP1A2) [103, 104]. This could also be a possibility for LMW (poly)phenol metabolites to distribute across the brain similarly.

Other types of transport may also be considered, especially in the disease context. These include vesicular transcytosis and paracellular transport that are strictly regulated in the BBB and are a less probable method for these types of compounds to reach the brain. Nevertheless, it has been proven that permeation through these two methods can be compromised in diseases within which inflammation occurs leading to an increase permeability of BBB to small molecules [105, 106].

There is still huge gap in knowledge about the fate of LMW (poly)phenol metabolites in the brain, especially due to the diversity of brain resident cells (Fig. 1). The fate of these LMW (poly)phenol metabolites should coincide with other xenobiotic molecules as well as endogenous molecules from the tyrosine or dopamine pathways. In the BBB and inside the brain, several phase I and II enzymes are known to be present. Phase I enzymes include CYP1B1, CYP2D6, CYP2E1, CYP2J2, CYP2U1 CYP46A1, CYP1B1 CYP1A1, CYP2U1, CYP3A5, CYP2R 1, CYP2E1, CYP2D6, capable of triggering (de)hydroxylation, (de)methylation and (de)alkylation reactions [107]. Moreover, a vast array of conjugation phase II enzymes can be found such as: glutathione-S-transferase 4 (GST4), glutathione S-transferase Mu 3(GSTM3), catechol-O-methyl transferase (COMT) and sulfotransferase1A4 (SULT1A4) [107]. Some studies have already demonstrated that some of these LMW (poly)phenol metabolites could undergo these types of metabolic reactions *in vitro* when incubated in the presence of the human brain microvascular cells [25]. Also, some catechin glucuronides were detected in the abluminal side in BBB models with RBE-4 and hCMEC/D3 cells, indicating the presence of UDP-glucuronyltransferases within the cells [43]. These types of metabolites could have an important function *in vivo*, yet much is awaiting to be discover regarding (poly)phenol metabolism in the brain.

## CONCLUSION

(Poly)phenol’s brain effects research reached a moment where it is crucial to embrace and clarify the discovery of which metabolites are reaching the brain. Several approaches have been used to understand this potential ranging from *in silico*, *in vitro* and *in vivo* methods. Understanding which are the metabolites from the dietary (poly)phenols that can in fact cross the BBB and reach the brain is imperative to further explore their potential benefits to the brain.

For some metabolites (4-hydroxybenzoic acid, vanillic acid, p-coumaric acid, ferulic acid, caffeic acid, protocatechuic acid) the *in silico* predictions of brain permeability were validated in *in vivo* studies. Also, the predicted inability to cross the BBB by sinapic acid could reflect its absence in the rat brain [51]. But this verification lacks for all metabolites. For example, SWISS ADME model seemed to predict gallic acid has not able to reach the brain by passive permeation, however it can be found in some experimental designs at relevant concentrations in the rat brain [51]. *In silico* models only reflect passive permeation and other possibilities should also be consider *in vivo*. Also the conclusions made from *in vivo* studies should be take with precaution, different conclusions about the percentage of brain permeability of (poly)phenols can be drawn if the proper interpretation is not made. There are many variables between studies regarding their experimental designs that influence the amount and type of LMW (poly)phenol metabolites detected in the brains that should be taken into consideration. The source of (poly)phenols is quite wide and variable and even the regular diet given to the rats can differ. Food complexity and interactions among the different components can affect the release of the compounds from the matrix and the uptake leading to different pharmacokinetic profiles. Moreover, the experimental design concerning the time of collecting the tissues for analysis after the last (poly)phenol administration will define the metabolites that will be detected and quantified in the brain. Additionally, as already highlighted, the rat strain, the concentration, the frequency and the route of the administration have also a strong influence on the amount and type of LMW (poly)phenol metabolites able to reach the brain. Sex differences may have also an important impact and it is a factor that most studies do not account. (Poly)phenols that target the brain are probably the physiologically active forms.

Understanding the mechanisms by which LMW (poly)phenol metabolites reach the brain will also impact the development of (poly)phenol derivatives as potential drugs with better pharmacokinetic properties. Another aspect, still in its infancy, is the knowledge on the potential enzymes and bio transformations that these compounds may be submitted to when entering the brain and once inside the brain. These further metabolic reactions may be decisive for their potential activity. Altogether, the presence and concentration in circulation, the percentage of crossing the BBB and the metabolism of LMW (poly)phenol metabolites is crucial if the objective is to design nutritional and pharmacological approaches to create a link between dietary factors and the prevention or treatment of neurodegenerative diseases.

## CONFLICT OF INTEREST

The authors declare no conflict of interest to report.
